# Molecular models of human P-glycoprotein in two different catalytic states

**DOI:** 10.1186/1472-6807-9-3

**Published:** 2009-01-22

**Authors:** Jean-Paul Becker, Grégoire Depret, Françoise Van Bambeke, Paul M Tulkens, Martine Prévost

**Affiliations:** 1 Structure et Fonction des Membranes Biologiques, Université Libre de Bruxelles, Boulevard du Triomphe CP 206/2, B-1050 Brussels, Belgium; 2 Unité de Pharmacologie cellulaire et moléculaire, Université catholique de Louvain, Avenue E. Mounier 73, B-1200 Brussels, Belgium

## Abstract

**Background:**

P-glycoprotein belongs to the family of ATP-binding cassette proteins which hydrolyze ATP to catalyse the translocation of their substrates through membranes. This protein extrudes a large range of components out of cells, especially therapeutic agents causing a phenomenon known as multidrug resistance. Because of its clinical interest, its activity and transport function have been largely characterized by various biochemical studies. In the absence of a high-resolution structure of P-glycoprotein, homology modeling is a useful tool to help interpretation of experimental data and potentially guide experimental studies.

**Results:**

We present here three-dimensional models of two different catalytic states of P-glycoprotein that were developed based on the crystal structures of two bacterial multidrug transporters. Our models are supported by a large body of biochemical data. Measured inter-residue distances correlate well with distances derived from cross-linking data. The nucleotide-free model features a large cavity detected in the protein core into which ligands of different size were successfully docked. The locations of docked ligands compare favorably with those suggested by drug binding site mutants.

**Conclusion:**

Our models can interpret the effects of several mutants in the nucleotide-binding domains (NBDs), within the transmembrane domains (TMDs) or at the NBD:TMD interface. The docking results suggest that the protein has multiple binding sites in agreement with experimental evidence. The nucleotide-bound models are exploited to propose different pathways of signal transmission upon ATP binding/hydrolysis which could lead to the elaboration of conformational changes needed for substrate translocation. We identified a cluster of aromatic residues located at the interface between the NBD and the TMD in opposite halves of the molecule which may contribute to this signal transmission.

Our models may characterize different steps in the catalytic cycle and may be important tools to understand the structure-function relationship of P-glycoprotein.

## Background

ATP-binding cassette (ABC) proteins form a large protein family in living organisms that carry various substrates across cell membranes. There are 48 ABC transporters in humans and mutations in many have been linked to genetic disorders[1,2].

The P-glycoprotein (P-gp), product of the *mdr1 *gene in humans, is one of these ABC transporters that extrudes a large range of structurally diverse compounds out of cells, a feature that has been described as poly-specificity[3,4]. It is the most extensively studied ABC transporter and can be used to understand the function of this important class of proteins. The overexpression of P-gp is associated with a multidrug resistance phenotype in various forms of cancer[5], which is a major barrier to the successful treatment of these diseases.

The vast majority of ABC transporters require energy in the form of ATP to translocate their ligands across the cell membrane. They require a minimum of four domains: two transmembrane domains (TMDs) form the ligand binding site and two nucleotide-binding domains (NBDs) bind and hydrolyze ATP to proceed to the substrate translocation. Two subtypes of ABC transporters can be defined on the basis of the direction of the transport reaction: ABC importers only present in prokaryotes and ABC exporters. Like many eukaryotic ABC transporters P-gp comprises all four domains in a single polypeptide chain. It is composed of 1280 residues, organized as two homologous halves (which are 43% identical in human P-gp) each with 6 transmembrane (TM) segments and a cytosolic NBD. Several helices of the TM domains have been proposed to accommodate the binding site(s) and consequently are believed to form the pathway through which the substrates cross the membrane. On the other hand the NBDs couple the energy associated with ATP binding and hydrolysis to drug transport[6]. The NBDs are better conserved than the TMDs and share several common motifs including the Walker A and B motifs that are found in other ATPases and the ABC signature that is unique to the family.

The P-gp mediated-transport mechanism has been extensively studied but remains controversial. Briefly it is believed[4,7] that the transport cycle is initiated by substrate binding in the TM domains of P-gp, which increases the ATP affinity for the protein. After binding and/or hydrolysis of a first ATP molecule in one of the NBDs several restructurings occur in the TM domains of the protein. This conformational change allows the release of the drug to the extracellular medium. P-gp returns to its original configuration possibly after hydrolysis of a second ATP molecule. The protein is then reset for another cycle. Two alternative models for the transport cycle of P-gp[7] are currently proposed and the most significant difference is in the nature of the mechanism that drives the drug from a high affinity site to a low affinity site in the TMDs. In one model[4] the formation of a closed NBD dimer provokes conformational changes that are transmitted to the drug binding site. Two sequential ATP hydrolysis events reset the P-gp molecule. Other models[8] require either one[9] or two[8] hydrolysis reactions to supply the efflux of the drug and the resetting of the protein for another cycle.

The considerable body of biochemical data obtained on ABC transporters has been more recently complemented by X-ray structures. At present several complete structures are available. All are of bacterial origin: five are ABC importers (BtuCD, ModBC-A, HI1470/1, MalFG/K, MetNI) [10-16] and two are drug exporters (SAV1866 and MsbA) [17-19]. In ABC importers the four domains exist as separate units whereas bacterial exporters are half transporters with one TMD and one NBD per polypeptide which function as a homo or hetero-dimer. These structures as well as genomic analysis and TM predictions[20] suggest that the domain organization of the importers is not shared by that of the exporters and that the TMD topology is also different.

Together biochemical data and structure determination can help in understanding the mechanism of the ABC exporters. However crystallization of membrane proteins to obtain direct information on three-dimensional (3D) structure is still a difficult task and no atomic-level structure of P-gp is available so far. Only structural information has been obtained at low resolution by electron microscopy (EM)[21,22]. In the absence of a high resolution structure for P-gp use of molecular modeling has been recommended in order to generate 3D models to sustain interpretation of experimental data[23] and potentially guide experimental studies. Several 3D models of the nucleotide-free protein were first published and most of them were either completely or remotely based on retracted former MsbA crystal structures[24]. Only two studies clearly stated that models constructed exclusively on MsbA dimeric structures were incompatible with cross-linking data on P-gp[25,26]. To unravel this problem, in one of these studies, the helices in the TMDs initially modeled from MsbA monomer structure were largely reshuffled using additional restrained potentials, defined from cross linking data, in molecular dynamics simulations[26]. However the construction process of this model lacked experimental data to properly describe the NBD:TMD interfaces and the relative position of the TM helices in each P-gp half.

The publication of the structures of SAV1866[17,18], which has a topology akin to that of P-gp prompted new 3D models of the nucleotide-bound conformation of P-gp to be constructed [27-29]. These structures have been determined in presence of ADP and AMP-PNP. They are very similar and feature a drug extrusion chamber open to the extracellular side. New crystallographic studies of ABC exporters trapped in different conformational states are needed to reveal changes along the catalytic cycle, f.i. upon substrate translocation and ATP hydrolysis. Revised MsbA structures trapped in different conformations have been very recently made available: two nucleotide-bound structures and two in absence of nucleotide[19]. The two nucleotide-bound structures possess an outward-facing conformation very similar to SAV1866 structures. The nucleotide-free structures determined at a quite low resolution feature two strongly different inward (intracellular)-facing conformations.

We present here four 3D models of P-gp describing two different states along the catalytic cycle using the recent X-ray structures of bacterial ABC exporters as templates. The models elaborated by comparative modeling are confronted to a large number of available experimental data. Interactions between conserved residues describing pathways from the ATP binding site towards the TMDs are examined to propose potential ways of transmitting a signal upon ATP binding or hydrolysis. Likewise conserved residues are pinpointed in the regions which could possibly be involved in the hinge binding motion suggested by the different crystallographic structures allowing the transition between the different conformational states. The interaction of compounds with P-gp is clearly a complex process. One of the nucleotide-free 3D models is used to dock several ligands in the central cavity harbored by the TM domains. The geometry of interaction of the ligands is examined and compared with the experimental data.

## Methods

### Selection of templates

A PSI-BLAST search[30] identified two bacterial ABC exporters homologous to human P-gp. One is SAV1866 from *S. aureus *whose structures were solved in complex to ADP[17] (PDB code 2HYD) and to AMP-PNP[18] (PDB code 2ONJ). The other is MsbA, a lipid flippase, which is available in two nucleotide-bound conformations (MsbA from *S. typhimurium; *PDB codes 3B60 and 3B5Z) and in two nucleotide-free conformations (MsbA from *E. coli *or *V. cholerae*; PDB codes 3B5W and 3B5X)[19]. The nucleotide-free structures include only the Cα positions.

### Sequence alignment

**Figure 1:**
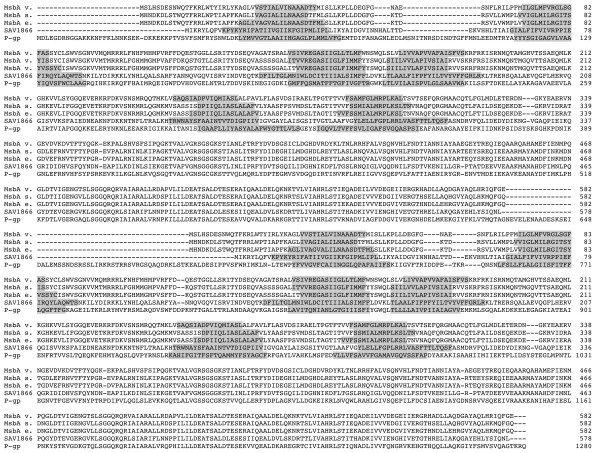
**Multiple sequence alignment**. Multiple sequence alignment of *V. cholera*, *S. typhimurium, E. coli *MsbA, SAV1866 and human P-gp used to generate the 3D models of P-gp. The predicted trans-membrane regions are grayed.

The sequences of human P-gp, *S. aureus *SAV1866, *V. cholerae *MsbA, *S. typhimurium *MsbA and *E. coli *MsbA can be accessed at the Universal Protein Ressource (UniProt). The percentage of sequence identity between P-gp and SAV1866 or MsbA ranges from 27% to 32% (sequence similarity is between 47% and 53%). Such percentages of identity make these templates potential candidates to elaborate 3D models of P-gp by comparative modeling. The similarity however varies noticeably for the cytosolic and transmembrane domains. The NBDs have sequence identity of about 50% and the TMDs have sequence identity ranging between 15% and 23% (sequence similarity between 30% and 43%). A multiple-sequence alignment of these protein sequences was performed using ClustalW[31] (see Figure 1).

### Trans-membrane domain prediction

Trans-membrane domain predictions were performed on the P-gp sequence using HMMTOP[32]. The results of these predictions which are reported on the ClustalW alignment (Figure 1) show a good correspondence with the trans-membrane segments of SAV1866 and MsbA determined with PDBTM[33].

### Model building

The multiple-sequence alignment was used as a basis to construct the models. It underwent only little modifications within two short sequence regions: one at the C-terminus of TM1 and the second at the residue pairs Asp498–Glu499 in the N-terminal half and Glu1143–Glu1144 in the C-terminal half. These changes were motivated by the sequence alignment generated from the structural superposition of the crystallographic structures of SAV1866 and *S. typhimurium *MsbA, in presence of AMP-PNP.

Four models describing P-gp in two different catalytic states were built using MODELLER 9v1[34]. Two models of P-gp in presence of nucleotides were constructed upon the structure of SAV1866 (PDB code: 2HYD) and the structure of *S. typhimurium *MsbA (PDB code: 3B60) respectively. Two models of P-gp in absence of nucleotide were built upon either the structure of *V. cholerae *MsbA (PDB code: 3B5X) or that of *E. coli *MsbA (PDB code: 3B5W). Each run produced ten different conformations that were optimized with the variable target function method employing methods of conjugate gradients and molecular dynamics with simulated annealing. Only the five models with the lowest potential energy were kept for further analysis. Measured distances were calculated on each model and averaged.

MODELLER builds models by satisfying different types of spatial restraints which include homology-derived restraints, stereochemical restraints obtained from CHARMM22 force field and statistical preferences for dihedral angles and non bonded distances obtained from a representative set of protein structures. Models are thus not constructed differently whether one resorts to all heavy atom or Cα atom only templates.

The positioning of one ATP molecule in each active site was performed as follows: the template structure either the SAV1866 or MsbA structure bound to AMP-PNP was superposed onto the corresponding 3D model. The nitrogen atom in AMP-PNP was changed into oxygen in a coordinate PDB file containing the 3D model. The sodium ion close to AMP-PNP in the SAV1866 structure was also changed into magnesium (the MsbA structure complexed to AMP-PNP contains no ion). Finally 100 steps of steepest descent minimization of the whole system were performed. The RMSD calculated before and after minimization is about 0.3 Å for ATP and 0.1 Å for all Cα atoms.

### Ligand docking

Docking was performed with the AutoDock program (version 4.00)[35]. Eight independent docking runs were carried out for each ligand starting from randomly generated initial conformations of the ligand. We used the genetic algorithm in AutoDock to perform the global search, completed with a local search, as it has been shown that it gives efficient sampling.

The binding zone was defined as a cube whose length was set to 30 Å. It enclosed the whole central cavity. The grid spacing was equal to 0.375 Å. The population size was set to 50 and the maximum number of generations to 27000. The rate of crossover was 80% and the maximum number of individuals that automatically survive was 1. The mutation rate was fixed to 2%.

The initial 3D structures of the ligands were generated with the CORINA program[36]. The computed Gasteiger[37] and Kollman united-atom atomic partial charges[38] were ascribed for the ligands and the protein respectively.

## Results

### Modeling of P-gp conformational states

In each model three P-gp fragments were not modeled because they lacked template. These three regions are: the first thirty N-terminal amino-acids, the first extracellular loop (ECL1) and the sixty amino-acids of the linker which connects the N-terminal to the C-terminal halves of P-gp. The alignment of various mammalian homologues of human P-gp reveals that the amino-acid sequences of these three regions are not chiefly conserved which suggests that they are not essential for the function of P-gp. This has been experimentally demonstrated for two of these three fragments. Deletions of the glycosylation sites located in ECL1 have shown that this region is not essential for the activity of P-gp as a multidrug exporter[39]. ECL1 was modeled as two helices in a model derived from an EM study[21]. However, because of the low resolution of the EM structure and of the weak helical propensity of this fragment observed in a secondary structure prediction, we decided not to model this loop. As for the linker region, mutation or deletion experiments have proven that only structural flexibility is required to ensure ATP hydrolysis and drug transport[40].

**Figure 2:**
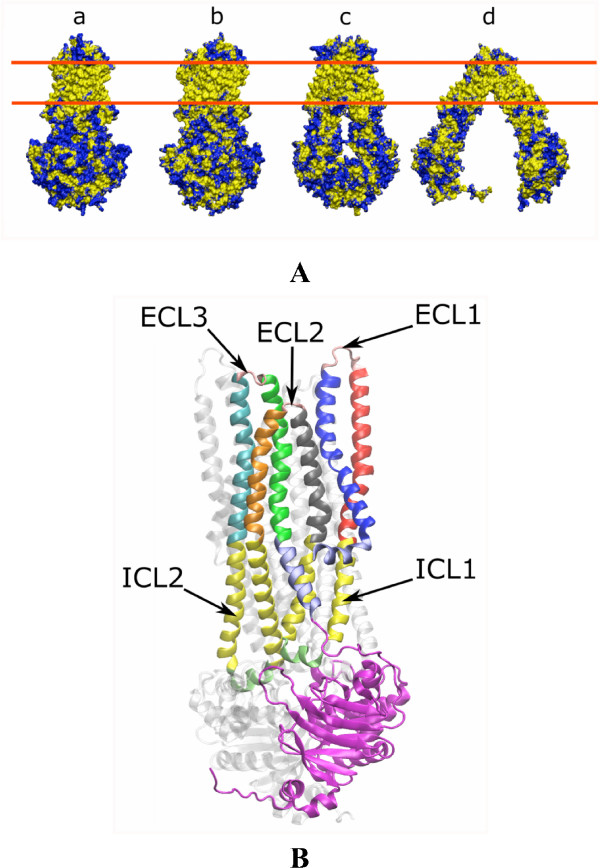
**A. Molecular surface representation of the 3D models**. (a) Nucleotide-bound model built with the SAV1866 template. (b) Nucleotide-bound model built with the *S. typhimurium *MsbA template. (c) Closed and (d) Open nucleotide-free models built with the *V. cholerae *MsbA and the *E. coli *MsbA templates respectively. The surface of hydrophobic residues (Ala, Leu, Val, Ile, Pro, Phe and Met) are colored in yellow. Other residues are colored in blue. The two red lines indicate the position of the lipid polar heads in the cellular membrane. **B. View of a nucleotide-bound conformation of P-gp**. The N-terminal half is highlighted: the three extracellular loops (ECL) are colored in pink (ECL1 is truncated (see text)), the two long intracellular loops are colored in yellow with the small coupling helices in pale green. The 6 trans-membrane helices are colored in blue (TM1), red (TM2), gray (TM3), orange (TM4), cyan (TM5) and green (TM6) and the nucleotide binding domain (NBD) is colored in magenta. The intracellular segment of TM1 and TM6 are depicted in light blue. All these segments are labeled from the N-terminus to the C-terminus.

Four models of P-gp (Figure 2) were built. Two models describe a nucleotide-bound state. One of the two models was built using as a template the structure of MsbA *S. typhimurium *complexed to AMP-PNP (PDB code: 3B60). In contrast to ATP, AMP-PNP can not be hydrolyzed thus MsbA has been trapped in a conformation that describes a state resulting from ATP binding. The other model was constructed using the SAV1866 structure determined in presence of ADP (PDB code: 2HYD). A more recent structure of SAV1866 in complex with AMP-PNP (PDB code: 2ONJ) shows no significant conformational change with the ADP-bound structure suggesting that this structure also mimics a post ATP-binding state. We used the ADP-bound structure of SAV1866 as a template because of its better resolution.

The other two models describe P-gp in different nucleotide-free conformations. One model was built using as a template the *Vibrio cholera *MsbA nucleotide-free structure (PDB code: 3B5W) and the other using the *E. Coli *nucleotide-free structure (PDB code: 3B5X).

The two nucleotide-bound models share the same outward-facing conformation whereas the two nucleotide-free models depict two different conformations: an open and a closed inward-facing conformation (see Figure 2).

The stereochemistry of the four models was assessed with PROCHECK[41]. The Ramachandran plots show a high percentage of the residues in the allowed regions: 99.7% and 98.3% for the nucleotide-bound models built with the SAV1866 and MsbA *S. typhimurium *structures respectively. 98% and 96.2% of the residues in the open and closed nucleotide-free models respectively are located in allowed regions of the Ramachandran plot.

The dimensions of the nucleotide-bound models are roughly 65 × 75 × 135 Å and the lengths of the TM helices range between 50 to 80 Å. The 12 TM helices exhibit a two-fold symmetry. These geometrical observations are consistent with the highest resolution images of P-gp bound to a nonhydrolyzable analog of ATP obtained by EM[21]. More recently, Lee *et al.*[22] have analyzed the structure of cysteine-free human P-gp from two-dimensional crystals in lipid monolayer in the presence or absence of various nucleotides. Their EM analysis predicts the nucleotide-free structure to be distinctly different from the nucleotide-bound one. The large conformational movement observed between our nucleotide-bound and nucleotide-free models agrees with these observations.

Figure 2A illustrates the molecular surface of the models colored according to the physico-chemical nature of the residues. Amino-acids contacting the lipid membrane form a hydrophobic belt round the TMDs. In contrast, the surfaces of the NBDs, the intracellular (ICLs) and extracellular (ECLs) loops are more hydrophilic. A significant number of aromatic (Tyr, Trp) and positively charged residues (Arg, Lys) are located at the borders of the membrane embedded region with their side chain oriented toward the outside of the structure. It has been proposed that these residues interact favorably with the polar heads of lipids[42]. This distribution of residues on the molecular surface of the models supports the quality of the alignments.

The structures of ABC exporters disclose that in contrast to ABC importers the TMD helices extend far beyond the membrane interface into the cytoplasm (see Figure 2B). Pertaining to these long ICLs are four short helices, also named coupling helices[43], roughly oriented parallel to the membrane that contact the NBDs. The striking feature that was revealed by the X-ray structure of SAV1866 and confirmed recently by the MsbA structures is that the second short helix in ICL2 and in ICL4 crosses over and associates with the NBD of the other monomer. The importance of this cross-over motif is even more highlighted in the two nucleotide-free structures of MsbA[19]. Cross-linking experiments performed between residues located in NBD1 and ICL4 or NBD2 and ICL2 [44,45] confirm the pertinence of this domain arrangement in P-gp (see Figure 2B). Our models are consistent with these data since the distances between the Cα of the residues involved in the cysteine mutagenesis range from 8.5 Å to 11.5 Å (see Table 1). Furthermore, the relative arrangement of ICL4 or ICL2 relative to the NBDs in our models agrees with several cross-linking data in CFTR[46], which shares the same topology and exhibits 36% of sequence similarity with P-gp. In CFTR, Phe508 of NBD1 can cross-link to several residues in ICL4 and Cys276 in ICL2 forms a cross-link with residues of the C-terminal NBD (see Table 1). The aligned sequences of the Q-loop regions and of these ICL portions feature about 50% similarity between P-gp and CFTR.

**Table 1:** Comparison of inter-residue Cα-Cα distances

Residues	Region	Cα-Cα distances (Å)				Exp. cross-linking (Å)	Ref.
				
		Nucl.-bound*	Nucl.-bound**	Closed-nucl.-free	Open-nucl.-free		
**NBDs**							
S1072/L531	WalkerA/Signature	**9.9**	**10.5**	41.0	65.6	5.5–15	[47]
S1072/S532		**6.8**	**7.5**	37.7	63.2		
G1073/L531		**7.6**	**8.3**	37.2	68.1		
G1073/S532		**5.4**	**6.3**	33.9	65.8		
G1073/G533		**7.9**	**8.9**	30.5	65.2		
C1074/L531		**10.8**	**10.8**	34.9	65.4		
G1075/L531		**10.4**	**10.6**	37.5	66.7		
S429/L1176		**10.0**	**10.6**	40.1	66.5		
G430/L1176		**7.6**	**8.4**	36.3	68.7		
G431/L1176		**10.9**	**10.9**	34.1	66.6		
G432/L1176		**10.5**	**10.7**	36.9	68.1		
C431/L1176		**10.9**	**10.9**	34.1	66.6		[48]
L531/C1074		**10.8**	**10.8**	34.9	65.4		
G427/C1074	WalkerA/WalkerA	31.0	30.9	**13.5**	56.8		[63]
L439/C1074		36.6	35.7	19.4	64.6		
C431/C1074		33.4	33.1	**7.9**	54.0		[64]

**NBD:TMD**							
L443/S909		**8.6**	**9.8**	**14.2**	**9.2**	6–16	[44]
S474/R905		**8.9**	**9.0**	**8.4**	**11.4**		
A266/F1086		**8.6**	**10.0**	**13.8**	**9.7**	5.5–15	[45]
							
Y490/V907 (CFTR: F508/L1065)		**7.9**	**9.1**	**12.5**	**9.2**		[46]
							
Y490/L910 (CFTR: F508/F1068)		**7.2**	**6.9**	**8.7**	**8.0**		
							
Y490/T911 (CFTR: F508/G1069)		**9.5**	**8.2**	**11.5**	**9.5**		
							
Y490/F916 (CFTR: F508/F1074)		**9.3**	**10.2**	**10.6**	**10.3**		
							
E493/T911 (CFTR: V510/G1069)		**10.6**	**8.6**	**8.3**	**10.0**		
							
V478/T906 (CFTR: W496/T1064)		**6.5**	**7.4**	**8.2**	**9.6**		
							
F480/V907 (CFTR: M498/L1065)		**10.8**	**10.8**	**11.7**	**11.7**		
							
R547/T911 (CFTR: K564/G1069)		**11.0**	**9.9**	**15.4**	**13.0**		
							
G269/Q1107 (CFTR: C276/Q1280)		**8.4**	**10.4**	**13.5**	**11.8**		
							
G269/A1111 (CFTR: C276/K1284)		**8.5**	**10.1**	**13.4**	**11.2**		

**TMDs**							
A80/T74 (MsbA: F56/F56)	TM2/TM8	33.0	34.5	22.8	**12.9**		[68]
							
M68/Y950	TM1/TM11	**9.5**	**11.8**	15.7	**10.8**		[94]
M68/Y953		**7.8**	**13.0**	16.9	**11.7**		
M68/A954		**8.1**	**12.8**	17.9	**13.5**		
M69/A954		**10.9**	**15.2**	20.8	**16.1**		
M69/F957		**10.3**	**15.4**	20.5	15.4		
V133/G939	TM2/TM11	**5.6**	**5.7**	**12.0**	**7.0**		[95]
C137/A935		**6.7**	**6.9**	**10.5**	**7.6**		
^117^YYS^119^/^555^GCF^557^#		**9.1–15.7**	**9.6–15.6**	16.5–22.0	**9.7–15.5**	6–16	[25]
S222/I868	TM4/TM10	41.4	46.1	42.1	34.3	9–25	[96]
S222/G872		41.7	45.0	41.5	34.3		
L227/S993	TM4/TM12	32.8	34.9	37.8	31.4	5.5–15	[97]
V231/S993		32.4	33.1	34.2	30.3		
W232/S993		29.4	29.9	30.7	28.5		
A233/S993		28.1	28.6	29.6	25.4		
I235/S993		30.9	30.9	30.6	29.7		
L236/S993		27.3	27.4	27.2	26.6		
N296/G774	TM5/TM8	**5.6**	**5.8**	**11.9**	**7.1**		[98]
I299/F770		**7.7**	**7.9**	**11.5**	**9.5**		
I299/G774		**8.8**	**10.3**	16.7	**11.9**		
G300/F770		**5.6**	**6.9**	**12.8**	**7.7**		
^317^GTT^319^/^753^NLF^755^#		**9.8–15.2**	**9.2–14.0**	19.0–23.8	**9.4–14.6**	6–16	[25]
I306/I868	TM5/TM10	36.3	41.0	34.6	32.1	13–25	[96]
I306/G872		36.7	40.4	34.9	33.4		
I306/T945	TM5/TM11	33.5	37.0	30.0	33.2		
I306/V982	TM5/TM12	**24.4**	31.3	**24.4**	**19.0**		
I306/G984		27.8	31.1	26.1	**21.5**		
A295/S993		23.2	24.1	24.2	22.3	5.5–15	[97]
I299/S993		22.6	24.5	26.3	21.2		
L339/F728	TM6/TM7	**6.7**	**10.7**	**18.1**	**19.2**	20–25	[99]
P350/V874	TM6/TM10	32.5	33.2	34.5	29.9	5.5–15	[97]
P350/E350		29.4	30.1	31.0	27.9		
P350/M876		28.2	28.6	29.7	24.8		
L339/I868		30.0	37.1	30.4	**23.3**	13–25	[96]
L339/G872		32.0	37.7	29.9	**23.7**		
L332/Q856		36.0	41.3	33.5	21.4	10–17	
P350/G939	TM6/TM11	22.9	23.6	25.3	19.2	5.5–15	[97]
L339/T945		27.5	33.1	**24.5**	**21.5**	20–25	[100]
L339/F942		26.7	31.2	**22.1**	**21.0**	25	[96]
L332/L975	TM6/TM12	21.9	27.3	17.6	**7.8**	5.5–15	[99]
F343/M986		16.4	20.0	18.6	21.6		[101]
G346/G989		20.2	23.7	23.5	26.4		
P350/S993		17.3	19.5	25.4	32.1		
F343/V982		16.8	23.3	20.5	18.9	10	[99]
L339/V982		**15.6**	**25.1**	**20.7**	**15.1**	16–25	
L339/A985		**19.6**	26.9	**23.2**	**19.1**	20–25	[96]
L332/L976		22.0	28.2	17.8	**11.2**	9–13	

Distances calculated in the nucleotide-bound (nucl.-bound) and nucleotide-free (nucl.-free) models are compared with distances derived from experimental cross-linking data determined in P-gp, CFTR and MsbA. Calculated distances in the range of the experimental distances are shown in bold and underlined. (*: SAV1866 template and **: *S. typhimurium *MsbA template; #: Cross-linking is observed for each residue of the triplet)

Table 1 compares the inter-residue distances measured in all four models and the experimental distances derived from cross-linking data. The measured distances between NBD:NBD and NBD:TMD residues are in better agreement than those involving TMD:TMD residue pairs. Some of the distances are comparable in the nucleotide-bound and the nucleotide-free structures whereas others are only in accord with data in one of the two states. The cross-linking distances tend to disagree more in studies probing the central cavity within the TMDs. This can be explained by the huge flexibility within P-gp and, in particular within its TMDs which may cause a potential problem in cross-linking studies. Indeed at room temperature cysteines may sometimes come closer and then crosslink even though they are relatively far apart in most of the sampled conformations. Measured distances between TM2–TM8, TM1–TM11, TM2–TM11, TM5–TM8, TM6–TM7 however agree reasonably well with experimental cross-linking data.

### Nucleotide-bound models

The two constructed models adopt very similar conformations (rmsd = 1.9 Å, calculated on the Cα superposed in an alignment of the structures) in agreement with the structural similarity of the two templates. The RMSD between the modeled P-gp structures and their corresponding templates is 0.5 and 0.3 Å for SAV1866 and MsbA structures respectively. The NBD dimer closes the cytoplasmic side of the trans-membrane domain. The bundle of trans-membrane helices is closely packed near the NBDs while it is wide-open to the extracellular space shaping an 'outward-facing' conformation[17]. We positioned two ATP molecules in each model using the location of the nucleotides in the crystallographic structures of their respective template (see Methods). This shows that the tightly bound NBD dimer occludes the two nucleotide molecules.

In both models, the nucleotide-binding pocket displays no opening that would allow entry of an ATP molecule from the solvent. Likewise, these nucleotide-bound models do not feature any potential entry through which substrates could enter the transporter either laterally from the membrane leaflet or from the cytosol. These findings suggest that conformational changes should occur to permit the access of the substrate and of the nucleotide molecule.

#### Structural interpretation of NBD mutants

**Figure 3:**
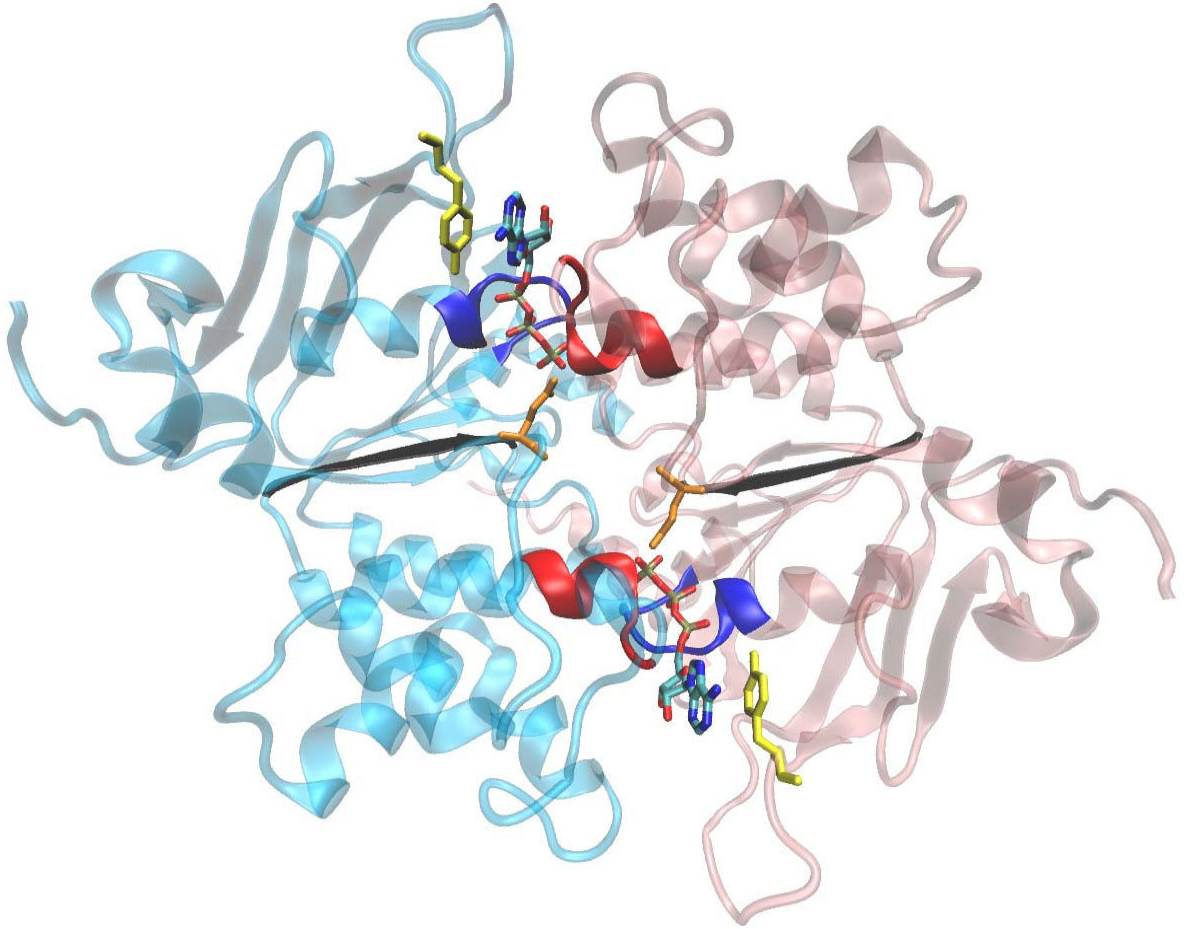
**NBD closed dimmer**. Ribbon representation (top view from the membrane) of the NBD closed dimer of one of the nucleotide-bound models in presence of two ATP molecules depicted as sticks. The N-terminal NBD (light blue) and the C-terminal NBD (light pink) are associated in a head to tail fashion. Each ATP binding pocket is formed by the A-loop (yellow), the Walker A motif (dark blue), the Walker B motif (black) and the Gln of the Q-loop depicted in orange from one NBD and is closed by the Signature motif colored in red from the other NBD.

In our models each ATP-binding site is formed by the residues of the Walker A, Walker B and Q-loop from one NBD and is closed by residues of the signature motif from the other NBD (Figure 3). Both NBDs are then associated in a head to tail fashion to form two binding pockets with the nucleotide sandwiched between the Walker A and the signature motif that face each other. This feature is sustained by cross-linking experiments (Table 1), which show that residues of WalkerA can be linked to residues of the signature in the opposite NBD[47,48].

Our models show that Lys433 and 1076 in the WalkerA motifs are located at a close distance of the ATP phosphates (the distance between the Nζ and Pβ or Pγ atoms is about 4.5 Å). This would explain that mutations of these lysine residues[49,50] disrupt ATP hydrolysis and even affect ATP binding to various degrees.

Tyr401 and Tyr1044 in NBD1 and NBD2, respectively, named A-loop[51], are well conserved and appear to be essential for ATP-binding[51,52]. Their equivalent tyrosine residues in hamster P-gp were shown to lie close to the adenine ring of a bound ATP in a photolabelling study[53]. In both models, each tyrosine forms a stacking interaction with the ATP adenine ring suggesting its importance for the nucleotide affinity.

The glutamate in the Walker B motif, often referred to as the 'catalytic carboxylate'[54], is also well conserved across species. In our models, Glu556 and Glu1201 are located near the ATP γ phosphate group (the distance between the Oε and the Pγ atoms is 5.1 Å.). Several mutations have demonstrated that both the chemical nature and the length of the side chain at these positions are essential for the binding and release of the nucleotide[55] as well as for the catalysis of hydrolysis[55]. Asp555 and Asp1200 are also part of the Walker B motifs. The mutation of these residues to asparagine abolishes both basal and drug stimulated ATP hydrolysis[56]. These two residues are also thought to participate to the Mg^2+ ^binding[57]. In our models, the side chains of Asp555 and Asp1200 are oriented toward the phosphate Pγ (The distance between the Oδ and the Pγ atoms is 6.6 Å).

The Q-loop[58] contains a well conserved glutamine residue at position 475 and 1118 in NBD1 and NBD2 respectively. It is located between the WalkerA and signature motifs in an otherwise less conserved region. In our models, the residues of the Q-loop participate both to the nucleotide binding and to the NBD:TMD interface. (the distance between the Oε and the Pγ atoms is 4.3 Å.). In P-gp, the most obvious effect of mutation of this glutamine is to reduce stimulation of ATP hydrolysis by drugs[59].

### Nucleotide-free models

Our two models show that the bundle of trans-membrane helices is closed on the extracellular side and open towards the cytosol (Figure 2A). These conformations feature an 'inward-facing' state. The RMSD between the modeled P-gp structures and their corresponding templates is 1.7 Å for the open nucleotide-free model and 0.5 Å for the closed nucleotide-free model. In the model built upon the closed nucleotide-free structure, the NBDs establish a loosely tight dimer, though they do not form an ATP sandwich as in the nucleotide-bound state. In the model built using the open nucleotide-free structure, the dimer is disrupted. The capacity for ABC exporters to sample a large conformational space is supported by spectroscopic data on LmrA[60], a structural bacterial homologue of P-gp that can functionally substitute P-gp and shares its substrate specificity[61]. High motional flexibility within the TM domain of LmrA was also reported by ATR-FTIR spectroscopy and ^1^H-^2^H exchange[62].

In contrast to the nucleotide-bound model the nucleotide-free conformations feature openings that would allow the entry of a nucleotide molecule to the active site. The closed nucleotide-free model depicts the two Walker A motifs facing each other. This feature is in agreement with cross-linking experiments[63,64] showing that Cys1074 in NBD2 can be cross-linked to Cys431, Gly427→Cys or Leu439→Cys in NBD1 (Table 1). The open nucleotide-free model portrays the two Walker A motifs at a distance of about 50 Å producing a wide entrance to the interior of the TM domain. This could allow the access of transported substrates, some of which are quite bulky. Such a large opening has been reported by EM structural studies on other ABC transporters like Mdl1[65] and YvcC[66]. More importantly a very recent ESR study on MsbA[67] reports that the closing of the chamber towards the cytoplasmic side occurs through very large movements and that the NBDs are very far apart in the absence of nucleotide as in the open MsbA X-ray structure. The open nucleotide-free structure is also consistent with a cross-linking distance between residues located at the extracellular side of TM1[68] (see Table 1).

#### Ligand binding

**Figure 4:**
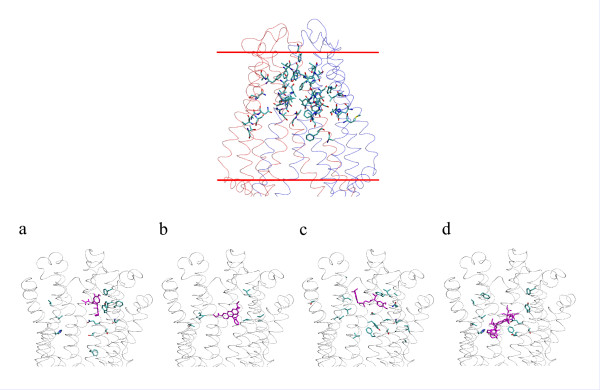
**Top: Drug binding site of the closed nucleotide-free model**. Tube representation of the transmembrane region of the closed nucleotide-free model. The residues experimentally identified to alter drug specificity are represented in balls and sticks: H61, G64, L65, Y118, V125, M197, T199, S222, I306, A311, V331, T333, F335, S337, V338, L339, I340, G341, A342, F343, Q725, F728, A729, S766, T769, I840, A841, N842, I864, I867, I868, A871, G872, A935, F938, F942, S943, T945, Q946, Y950, F951, S952, Y953, F957, L975, F978, V981, V982, F983, G984 and A985. The two red lines indicate the position of the lipid polar heads in the cellular membrane. **Bottom: One predicted position for each docked ligand**. Ligands are depicted in purple and the residues experimentally identified to alter their specificity are colored according to their chemical type (carbon in cyan; oxygen in red; nitrogen in blue). (a) colchicine, (b) rhodamineB, (c) verapamil and (d) vinblastine.

One of the intriguing features of P-gp is that it recognizes and transports a large variety of substrates. Though numerous studies have attempted to identify mutations in P-gp that affect the recognition and transport of substrates the localization of the binding site(s) is still an open question. Here we use the closed nucleotide-free model to locate residues known to be involved in drug binding. Strikingly all residues which have been identified as affecting the drug specificity [69-81] are confined mainly in the TM domain embedded in the outer membrane leaflet (see Figure 4). Almost all residues face the large central cavity. A few of these facing residues however are shielded from the central pore. In particular, Ser222 in TM4 is hidden by TM5 and TM6 and Ile868, Ala871 and Gly872 of TM10 are shielded by TM11 and TM12.

In order to further assess the accuracy of the model we performed the docking of a number of P-gp ligands. The scope is double: first to verify whether the central cavity can accommodate the different ligands and second to analyze the binding site for each ligand and identify the contacted residues. Docking was carried out on verapamil, rhodamineB, colchicine and vinblastine, which differ by their size, topology and their chemical functionalities.

The volume in which the ligands were docked was centered on the geometric center of the residues experimentally identified to alter drug specificity (see listed residues in the legend of Figure 4). This volume was taken large enough to enclose the large central cavity that runs between the borders with the cytosol and the extracellular medium. Nevertheless most of the positions of the different ligands are found in the TM domain located in the outer membrane leaflet portion. Vinblastine however which is the biggest of the docked ligands occupies a region which runs from the outer leaflet to the middle part of the inner leaflet and one of the docked poses of rhodamineB is found closer to the TM domain located in the inner leaflet. Interestingly all docked poses for each ligand are found to interact with residues which have been experimentally identified to bind a specific ligand (Figure 4 and Table 2). The docked poses for verapamil roughly sample two locations, one of which presents several contacts with experimental binding residues. The docked positions of rhodamineB are the most spread of all ligands with however one more populated cluster. Colchicine exhibits two groups of poses one of which, most populated, makes interactions with experimentally identified residues. The poses of vinblastine cluster mainly into one group. Each pose fills almost completely the upper part of the central cavity which narrows towards the extracellular side. The drug binding site in our model shows that none of the ligands because of their size and of the distance between the binding site residues can bind at the same time all its experimentally identified residues (Figure 4).

**Table 2:** Drug binding site residues

Pose	1	2	3	4	5	6	7	8
Colchicine	**Gln946**	**Gln946**	Ile306	Ile306	Ile306	Ile306	Ile306	Leu339
	**Tyr950**	**Tyr950**	**Phe335**	**Phe335**	**Phe335**	**Phe335**	**Phe335**	Gln725
	**Tyr953**	**Tyr953**	Phe728	Phe728	Leu339	Phe728	Phe728	Phe728
	**Phe957**	**Phe957**			Phe728			Phe983
		Leu975						

RhodamineB	**Leu65**	His61	Phe728	Ile306	Ile306	Ile306	Ala311	Gln725
	**Ile340**	**Leu65**	Tyr950	Phe335	Phe335	Phe728	Phe335	Phe728
	Phe343	Val125	Tyr953	Phe728	Phe728	Phe983	Phe728	Phe983
	Phe942	Phe938	Phe957	Phe983	Phe983			
	Tyr950		**Leu975**					
	Tyr953							
	Phe957							
	Val982							

Verapamil	**Ile306**	**Ile306**	**Ile306**	**Ala311**	**Leu65**	**Ile306**	**Ile306**	Leu975
	Phe335	Leu339	Phe335	Phe335	Val125	Phe335	Phe335	Thr333
	Leu339	Phe343	**Phe728**	**Phe728**	**Phe942**	Leu339	Leu339	
	**Gln725**	**Gln725**		Leu975		Phe345		
	**Phe728**	**Phe728**						

	Val125	Ile306	Val125	Val125	**Leu65**	Ile306	Ile306	Phe343
	Ile340	**Phe335**	Leu339	Leu339	Ile340	Thr333	**Phe335**	Gln725
	Phe343	Leu339	Ile340	Ile340	Phe343	Phe335	Leu339	Phe728
Vinblastine	**Phe942**	Ile340	Phe343	Phe343	Phe728	Leu339	Phe343	Ser766
	**Gln946**	Phe343	Phe938	**Phe942**	Gln946	Ile340	Phe728	Phe983
	Val982	Phe728	**Phe942**	Val982	Phe983	Phe728	Phe983	
	Phe983	Phe983	Phe983	Phe983		Phe983		

Residues found to interact with each of the 8 predicted positions of the four different ligands (see text) docked in the central cavity of the closed nucleotide-free structure. The residues experimentally identified to alter the specificity of a particular ligand are reported in bold and underlined.

### Conformational changes: signal transmission and triggering

Binding and/or hydrolysis of ATP induce conformational changes that are transmitted from the NBDs to the TMDs. The nature and amplitude of these conformational changes remain elusive though the recent X-ray structures of ABC exporters trapped in different states together with the numerous biochemical data[82] suggest a possible role of different portions of the protein in the transmission of a signal following ATP binding.

In this section, we analyze our models to propose potential transmission pathways produced by ATP binding and/or hydrolysis. The residues involved in the transmission mechanism of ABC exporters are likely to be conserved across species. So to isolate them, the amino-acid sequence of each P-gp half was aligned separately with the sequences of 200 homologous ABC exporters using ConSurf[83].

Starting from the nucleotide-bound models, so as to take advantage of the presence of a nucleotide molecule in each binding pocket, we analyzed the interactions made by each nucleotide with neighboring residues identified by ConSurf as conserved residues having a potential functional role. In what follows we describe the potential pathways starting from the N-terminal and the C-terminal NBDs. For sake of clarity, the residues and protein fragments identified starting from the C-terminal NDB are given in curly brackets.

**Figure 5:**
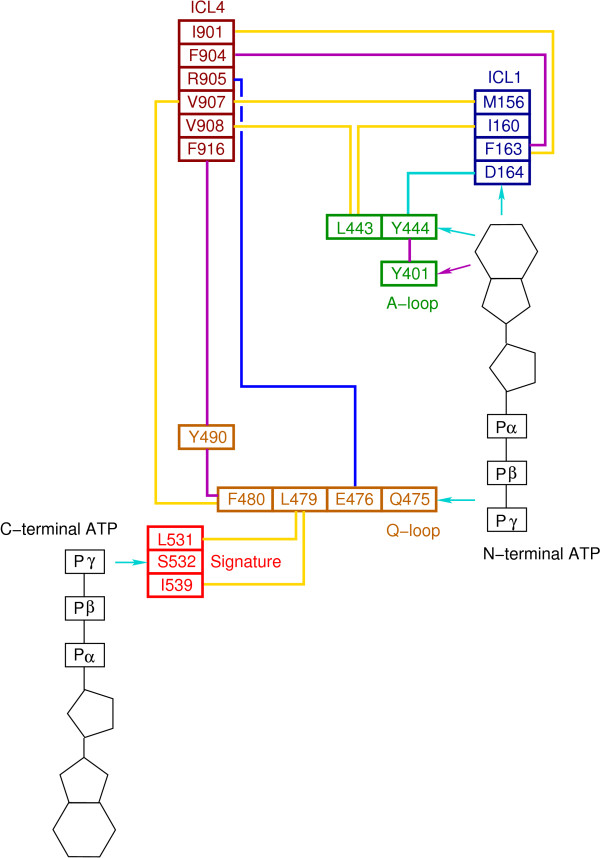
**NBD:TMD communication pathways**. For sake of clarity only the residues identified starting from the N-terminal NBD are indicated. First pathway: From adenine → directly or through NBD aromatic residues → ICL1 → ICL4. Second pathway: From ATP phosphates → directly to Q-loop of the same NBD or through ABC signature from the ATP of the facing NBD to the Q-loop → ICL4. The colors of the links between the residues depict the physical nature of their interactions: yellow: hydrophobic, magenta: aromatic-aromatic, cyan: hydrogen bond and blue: electrostatic.

We detected two main ways of transmission, which could originate from residues interacting either with adenine or with the γ phosphate group of ATP (Figure 5).

In both the N- and C-terminal halves, the adenine makes a hydrogen bond with a conserved Asp164 {Asp805} of the small coupling helix of ICL1 {ICL3}. Transmission could also arise through the interactions of adenine with Tyr444 {Tyr1087} whose side chain hydrogen bonds both to adenine and to Asp164 of ICL1 {Asp805 of ICL3}. Furthermore, Tyr444 {Tyr1087} makes an edge-to-face interaction with Tyr401 {Tyr1044}, the A-loop, strongly conserved across species, which, itself, stacks to the adenine. Interestingly Leu443 {Phe1086} preceding Tyr444 {Tyr1087} interacts directly with residues of ICL1 (Ile160) {ICL3 (Val801)} and residues of ICL4 (Val 908) {ICL2 (Ile265)}. Moreover, a cation-π interaction is likely to occur between Phe1086 and Arg262 of ICL2. This last interaction has no equivalent in the N-terminal NBD. Propagation of the signal from the residues in ICL1 {ICL3} could then occur through contacts with residues of ICL4 {ICL2}. In particular Phe163 {Phe804} forms a hydrophobic cluster with residues of ICL4 (Ile901 and Phe904) {ICL2 (Leu258 and Ile261)}. Other hydrophobic contacts are also formed between Met156 in ICL1 {Leu797 in ICL3} and Val907 in ICL4 {Val264 in ICL2}. This pathway can be summarized as follows: adenine interacts either directly or through nearby aromatic residues of the NBDs with the coupling helix in ICL1 {ICL3} which itself contacts residues in ICL4 {ICL2}. This transmission strongly implies Asp164 as an important actor. Asp164 pinpointed by ConSurf is indeed conserved in MsbA. However it is replaced by an alanine in SAV1866. A look at the SAV1866 structures does not disclose which neighboring residue could substitute for Asp164 as a hydrogen bond maker or which other interaction could replace the ones observed between Asp164 and either adenine or NBD residues.

Another pathway for signal transmission following ATP binding could originate from the interaction of the γ phosphate group with Gln475 {Gln1118} of the Q-loop. In the same loop, Phe480 {Phe1123} makes hydrophobic interactions with Val907 {Val264} located in the middle of the coupling helix in ICL4 {ICL2}. Interestingly, at the NBD:TMD interface, Phe480 {Phe1123} shapes an aromatic cluster with Tyr490 {Tyr1133} and Phe916 of ICL4 {Phe267 of ICL2}. The equivalent residue of Phe916 in the N-terminal half is substituted by Glu273 in ICL2 but another aromatic residue, Phe267 within ICL2 fills in the aromatic cluster in the model built using MsbA structure. In the model based upon SAV1866, the orientation of Phe267 differs, such that no triple aromatic interaction is observed with the other two residues (Phe1123 and Tyr1133). This may be due to the non-conservation in SAV1866 of the aromaticity at that position (Figure 1). Deletion of Tyr490 is known to induce defective or no expression of P-gp[84] and the deletion of the equivalent residue of Tyr490 in CFTR, Phe508, is the major cystic fibrosis causing mutation[85]. Remarkably an equivalent aromatic cluster was identified in a 3D model of CFTR and its functional importance was reported[46]. In some models an electrostatic interaction was also observed between Glu476 of the Q-loop {Glu1119} and Arg905 {Arg262} in ICL4.

Interestingly Phe480 {Phe1123} of the Q-loop is also preceded by a conserved leucine, which interacts with residues of the ABC signature: Leu531 and Ile539 {Leu1176 and Ile1184} which close the ATP binding pocket of the other NBD.

The second pathway can be summarized as follows: ATP phosphates interact with residues of the Q-loop which itself contacts residues of the coupling helix in ICL4 {ICL2}. In a more complex way the signal to ICL4 {ICL2} in one NBD could also originate from the phosphates of the facing NBD through residues of the ABC signature which itself contacts the Q-loop.

Both proposed pathways emphasize the importance of ICL4 {ICL2} and to a lesser extent ICL1 {ICL3} in the transmission of a signal from the ATP binding pocket. The ICL4 {ICL2} links the TM segments (either TM4–TM5 or TM10–TM11) which mediate the hinge binding motion allowing the transition from the inward to the outward conformations as suggested by the MsbA structures. Remarkably it was shown that CFTR mutants located in the coupling helix in ICL4 displayed a decreased channel open probability suggesting the importance of dynamic contacts at these sites for the conformational change to occur in this protein[46,86,87]. Also mutations in ICL1 of CFTR were reported to impede transition to the open state of the protein[88].

**Figure 6:**
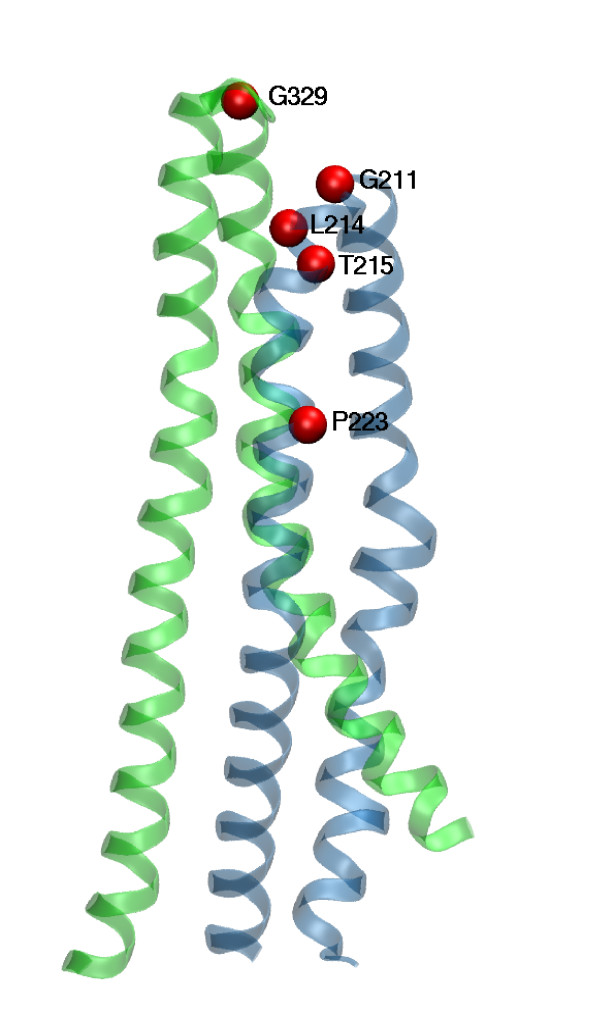
**Residues potentially involved in the hinge bending motion of TM3–TM4 and TM5–TM6 pairs**. Ribbon representation of TM3–TM4 (in blue) and TM5–TM6 (in green). The conserved residues identified by ConSurf (see text) with a potential role in the hinge conformational change upon ATP binding are depicted as red spheres.

Comparison of the MsbA nucleotide-free and nucleotide-bound structures suggests that ABC exporters undergo large conformational changes. These structures pinpoint to a large motion of the portion formed by TM4/ICL2/TM5 {or TM10/ICL4/TM11} around a hinge defined by ECL2/ECL3 {or ECL5/ECL6}[19]. We looked at the conserved residues identified by ConSurf in these potential hinge regions. The most conserved residues are Leu214, Thr215 and Leu216 {Leu 857, Thr858 and Leu859} located at the N-terminal side of TM4 {TM10} and thus close to the extracellular loop between TM3 and TM4 {TM9 and TM10} (Figure 6). Interestingly it has been proposed that threonine or serine residues can cause local alterations which may result in significant conformational changes across transmembrane helices and which may play a role in transmembrane signaling[89]. Pro223 {Pro866} located further down TM4 {TM10} has also been identified as a conserved residue with a potential role. Despite their disruptive nature, proline residues are statistically well represented in transmembrane helices. Proline was reported to play an important role in producing conformational changes essential for receptor signaling and channel gating[90,91].

Studies[70,92] demonstrated that several residues in TM6 in particular those located at the two extremes show large changes in accessibility to covalent modification by maleimide reagents suggesting that TM6 undergoes significant conformational changes upon the catalytic cycle. Our nucleotide-free and -bound models can readily explain most of these site-directed labeling data. However, instead of pointing to a large TM6 motion, our models feature structural changes in TM6 neighborhood caused by the hinge bending motion of TM4–TM5 pair which swings away from TM6 and by the shift of TM7.

## Discussion and Conclusion

The lack of a high resolution structure for P-gp and the recently determined X-ray structures of several ABC exporters trapped in different conditions prompted us to construct several 3D models of P-gp featuring different states along its catalytic cycle. Sequence identity and similarities, though varying markedly across the protein, as well as experimental data support that SAV1866 and MsbA share the same architecture as P-gp.

We present here two nucleotide-bound and two nucleotide-free models of P-gp based on four different template structures. The two nucleotide-bound models which depict an outward-facing conformation of P-gp are very similar and are in good agreement with the 8 Å resolution EM data obtained on P-gp. The two nucleotide-free models which both portray an inward-facing conformation differ markedly by their opening towards the cytoplasm and by the absence of a NBD:NBD interface in the open model. EM and ESR data on homologous proteins also corroborate the occurrence of large conformational motions upon nucleotide binding.

All four models were carefully analyzed to compare modeled residue-residue distances to those estimated in cross-linking experimental studies obtained in a range of conditions: in the presence or absence of substrates, nucleotides and with chemical cross-linkers (see Table 1). Overall the pattern of distances from all four models agrees with these experimental data.

A large number of site-directed and scanning mutagenesis studies have been performed on P-gp to get insight into the molecular mechanism and to spot the residues essential for drug binding and translocation, ATP binding and hydrolysis. We showed here that our models can be used to rationalize the effects of several mutants in the NBDs, or at the interface NBD:TMD or within the TMDs.

Almost all residues known to affect the specificity of individual substrates strikingly face the inside of a large pore and are located mainly in the outer membrane leaflet. The closed nucleotide-free model can accommodate different ligands of different size. The mode of association observed for the docked ligands favors the existence of multiple binding sites within the large central cavity, a feature supported by several experimental studies. For each ligand, several positions are found to involve interactions with residues identified to alter drug binding (see Table 2).

Docking experiments (data not shown) indicate that no access is large enough to allow the entry of one ATP molecule into the catalytic site of the nucleotide-bound models suggesting that these structures should undergo changes to accommodate their ligands. In both nucleotide-free models ATP can enter to reach a site formed by Walker A and Walker B motifs which are the hallmark of some ATPases. However in the closed nucleotide-free structure the site formed by the Walker A and B motifs of one NBD is protected partially from the solvent by the other NBD and is likely to harbor only one ATP molecule. In the open nucleotide-free model two ATP molecules could possibly bind as each site formed by the Walker motifs is fully solvent-exposed. In that respect the latter model agrees with experiments showing that each P-gp half expressed in cells exhibits basal ATPase activity that cannot be stimulated by drugs[93]. One cannot however exclude that this activity could be due to the presence of homodimers formed by half molecules.

In contrast to the nucleotide-bound structures, both nucleotide-free models show possible entries for the substrates either laterally from the inner membrane leaflet or from the cytoplasm. The open nucleotide-free structure adopts a rather unique shape which discloses an unusual chamber within the TMD and a large space between the NBDs. These features are corroborated by quite recent ESR studies on MsbA. This structure also reveals entries large enough to allow access of the bulkier ligands of P-gp. The closed nucleotide-free structure is also sustained by several experimental data. It is the only structure to agree with the Walker A-Walker A cross-linking distance. This closed nucleotide-free structure could possibly either occur sequentially along the catalytic cycle after ligands enter the open nucleotide-free structure or coexist with the open structure as suggested by the high mobility noted by ATR-FTIR and NMR for LmrA[60,62]. The outward facing conformation of the two nucleotide-bound models features a central pore open to the extracellular medium that would allow substrates to escape.

The determination of 3D models at different stages of the catalytic cycle can help in proposing a role, in particular, of the residues essential for the transmission of a signal producing conformational changes or responsible for the conformational changes themselves. We indeed identified two potential pathways formed by a chain of interacting residues which could be involved in the propagation of a signal upon ATP binding from the catalytic site throughout to the TMDs. One highlighted a pathway describing contacts between adenine either directly or through aromatic residues with the coupling helix in ICL1 {or ICL3} which itself interacts with residues of ICL4 {or ICL2}. The other pathway depicts a chain of interactions starting from the ATP phosphate groups to ICL4 {or ICL2} by means of the Q-loop. One of the interaction sites involves a cluster of aromatic residues including residues of the Q-loop and of ICL4 or ICL2. Interestingly an equivalent cluster of aromatic residues was detected in CFTR and its role on the channel gating was revealed. Mutagenesis of only one glutamine of the Q-loop has been shown to inhibit the function suggesting that this mutant also affects the ATP binding/or hydrolysis in the neighboring NBD [59]. In that respect the second pathway may explain this observation as it incorporates residues which have been suggested to play a role either in the NBD:TMD communication (residues of the Q-loop) or in the NBD dimerization (signature motif). It should also be mentioned that several pathways either in series or in parallel could occur to transmit the signal from the active site to the TMD.

Our models are thus first approximation models and may constitute a useful starting point for the understanding of the complete structural picture of P-gp at the different stages of the catalytic cycle. They may guide further investigations of the role of residues at the NBD:NBD and NBD:TMD interfaces.

## Authors' contributions

GD constructed the P-gp model based on the Sav1866 template and participated in the elaboration of a coherent collection of cross-linking and mutagenesis data. J-PB constructed the other three models. J-PB and MP performed the structural analysis and the interpretation of the mutagenesis data using all models of P-gp. FVB and PMT participated in the design of the study. J-PB and MP wrote the manuscript. All authors read and approved the final manuscript.
